# Exploring Perceptions of Physical Activity in Individuals Newly Diagnosed with Multiple Sclerosis

**DOI:** 10.3390/jcm14041199

**Published:** 2025-02-12

**Authors:** Michael VanNostrand, David A. Henning, Lori Quinn, Andre Cabalang, Nora E. Fritz

**Affiliations:** 1 Department of Health Care Sciences, Wayne State University, Detroit, MI 48201, USAnora.fritz@wayne.edu (N.E.F.); 2 Department of Biobehavioral Sciences, Teachers College, Columbia University, New York, NY 11032, USA; lq2165@tc.columbia.edu; 3 Department of Neurology, Wayne State University, Detroit, MI 48201, USA

**Keywords:** multiple sclerosis, newly diagnosed, physical activity, barriers

## Abstract

**Background:** Physical activity is essential in enhancing the quality of life for individuals with multiple sclerosis (MS). However, there is limited evidence regarding the unique barriers individuals newly diagnosed with MS face. The purpose of this qualitative study is to understand the perspectives of persons newly diagnosed with MS about physical activity. **Methods:** Four focus groups were conducted with persons newly diagnosed with MS (*n* = 12). Participants were asked open-ended questions related to barriers and facilitators of physical activity, knowledge surrounding physical activity for individuals with MS they would find helpful, and how best to receive this information and track their progress. **Results:** Four themes were generated from the data: (1) lack of knowledge about MS leads to fear and physical activity aversion, (2) reimagining physical activity leads to frustration, (3) navigating physical activity constraints in a busy world, and (4) accountability is key to maintaining physical activity in the presence of barriers. The findings of the study were used to adapt an existing coaching intervention model to increase physical activity engagement specifically in persons with MS soon after the diagnosis. **Conclusions:** This study underscores the distinct challenges encountered by individuals newly diagnosed with MS, most notably the time constraints imposed by symptoms and employment commitments. These findings highlight the necessity of developing a tailored physical activity coaching intervention, such as ENGAGE-MS, that prioritizes education, accessibility, and adaptability to maximize benefits and develop long-term, sustainable physical activity behaviors.

## 1. Introduction

Multiple sclerosis (MS) is characterized by axonal demyelination within the central nervous system, resulting in fatigue, muscle weakness, sensory changes, and difficulty walking [1]. While the pathogenesis and progression of MS remain subjects of ongoing research, one area of growing interest and importance is the role of physical activity (PA) in managing the disease [2,3]. Previous research has demonstrated that increased levels of PA play a crucial role in symptom management, enhancing cardiovascular function, and promoting motor recovery in individuals with MS [4]. Despite the recognized benefits of PA for individuals with MS, current evidence suggests that they do not meet the recommended guidelines for the general population and are less physically active than non-diseased populations [5,6].

The consequences of low levels of PA in individuals with MS are far-reaching. Specifically, reduced PA has been associated with lower levels of community participation [7], increased risk of falls [8,9], fear of falls [10], loss of functional independence [11], and ultimately, a reduced overall quality of life [3]. While these findings underscore the importance of promoting increased PA at the time of the diagnosis, current MS treatment rarely includes increased PA promotion; instead, treatment primarily focuses on pharmaceutical disease-modifying therapies (DMTs) [12]. Consequently, referrals to community therapists are typically made only in the presence of significant gait and balance impairments [13]. However, early exercise and PA have been shown to effectively improve symptoms and slow MS-related decline, reinforcing the concept of “exercise as medicine.” [14]. As such, there is a critical gap in both education about and engagement in regular exercise among individuals newly diagnosed with MS.

The importance of increasing PA soon after the diagnosis is further emphasized by the exercise-induced postponement theory [14]. This theory postulates that long-term PA can postpone or delay the worsening of disease activity and symptoms in individuals with MS compared to the traditional course of MS. While this is true, current interventions to increase PA often overlook the importance of timing [15], with no specific interventions focused on individuals newly diagnosed with MS (i.e., diagnosed within the last five years). Notably, across the 65 intervention studies included in a recent review, the lowest average disease duration at baseline for an exercise intervention group was 4.9 years [16]. More recently, a feasibility study on a physical activity intervention for individuals newly diagnosed with MS demonstrated significant improvements in self-reported physical activity, light physical activity, and health-related quality of life [17]. Given the significance of early MS treatment and the potential of PA as a disease-modifying treatment [18], it is critical to incorporate and increase PA during this “window of opportunity” soon after the diagnosis to improve the long-term prognosis.

Several common barriers can significantly hinder regular engagement in PA for individuals with MS, including costs [19,20,21], transportation difficulties [20,21], limited local specialist services [22], fatigue [2,23,24], cognition [25], and a lack of confidence in exercise knowledge [26]. These findings align with a review of qualitative research that examined determinants and consequences of PA, which found that individuals with MS often cited lack of accessibility, conflicting advice from healthcare professionals, fatigue, fear, and apprehension as factors limiting exercise adherence [2]. While these findings were instrumental in understanding the causes of limited PA in individuals with MS, the articles included in this review did not focus on the barriers and facilitators faced by individuals with MS who are newly diagnosed. These individuals may have different lived experiences due to their lower disease severity and increased likelihood of being employed [27]. Furthermore, while previous qualitative research involving individuals newly diagnosed with MS has been conducted, it has predominantly focused on physical activity behavior change rather than exploring perceptions and barriers to physical activity [28,29]. Consequently, it remains unclear whether individuals who are newly diagnosed with MS experience similar barriers to PA engagement. This group may have unique barriers and facilitators that would be important to target with PA interventions. Therefore, we conducted a qualitative study to determine perceptions of PA, as well as specific barriers, facilitators, and needs of newly diagnosed individuals with MS. Finally, we incorporated the findings of this study into the development of a new PA coaching intervention aimed at addressing these unique challenges.

## 2. Methods

Individuals between the ages of 18 and 75 years old were included in the study. Prospective participants were considered newly diagnosed if they reported being diagnosed within the last five years, as the previous literature used this cut-off [30,31]. There were no exclusion criteria. Recruitment was conducted via social media postings from the National MS Society, flyers, and emails to local support groups, and from a registry of participants who have participated in prior studies of the laboratory and consented to being contacted. All study methods were approved by the Wayne State University Institutional Review Board (IRB), and all participants provided informed consent prior to participation in the study, which provided important details related to the psychological risks associated with a recent MS diagnosis.

A total of four focus groups were organized and facilitated by a physical therapist or postdoctoral research fellow specializing in MS (NF/MV), both of whom have prior experience with qualitative research [25,32,33], with a research team member present to observe and summarize the data. Focus groups were used to create a less formal atmosphere, ensuring that all participants would feel comfortable sharing their perspectives. This approach also enabled the facilitator to connect ideas between participants, fostering a more fluid conversation. The focus group questions (see Appendix A) were developed based on the current literature and the investigators’ prior research, adhering to established guidelines for formulating focus group questions [34]. This approach ensured a systematic and robust method of data collection. The formation of focus groups was terminated after four sessions, as discussions became repetitive, resulting in little to no changes in the themes, indicating that saturation had been reached [35].

During the focus groups, after each individual was introduced, participants were asked a series of open-ended questions concerning exercise (as outlined in Appendix A). These questions explored barriers and facilitators to PA, the information provided about PA at the time of the diagnosis, and preferences regarding the receipt of PA-related information. The focus group sessions typically lasted approximately one hour on Zoom (Zoom, CA, USA) and were audio-recorded and transcribed verbatim, with any identifiable data removed.

Following methods used in similar studies [36,37], codes, and categories (also referred to as themes) were defined using a combination approach of both inductive (open coding) and deductive (based on the prior literature) coding. The research team a priori-identified three expected themes: lack of knowledge/literacy of exercise, positive or negative perceptions of PA, and strategies to improve engagement in PA. After coding participant responses, a fourth theme was identified related to barriers to PA. Participant responses were coded such that each code related to one of the four themes. A short sentence was then used to generally describe the code, where appropriate direct quotes from participants were used to highlight the exact language or concern. Developing codes through open coding of the transcripts required judgements on meaning, importance, and connections between ideas. Two researchers (DH and NF) double-coded the transcripts to ensure that the same concepts about the data were expressed. Discussion led to renaming a number of codes and condensing several codes into a single code until 100% agreement was reached. Finally, the codes and categories indexed in the initial analytical framework were used to develop key themes.

## 3. Results

Participant demographics are presented in Table 1. Mean participant age was 52.67 ± 10.18 years and 3.08 ± 1.31 years post the diagnosis.

Four key themes were identified. A common finding across themes was that barriers to PA are pervasive; thus, it is critical that programs aiming to enhance PA engagement for persons with MS address barriers that may impact engagement and adherence. Furthermore, despite the heterogeneity within the group regarding disease severity (PDDS), age, and regular exercise, perceived barriers and facilitators remained consistent.

### 3.1. Theme 1: Lack of Knowledge About MS Leads to Fear and PA Aversion

Participants frequently expressed their lack of knowledge regarding when to initiate PA following their MS diagnosis. This uncertainty often gave rise to anxiety and, in some cases, outright fear of exacerbating their condition through exercise. One participant candidly shared the following:

*“I guess how soon you should start. Like when you first have an attack, you’re knocked down, and that’s the last thing you want to do. But my doctor was writing me a referral for physical therapy right away, and I was like, ’Are you kidding me? I don’t even want anyone near me.’ Whatever is going on with my body from the waist down, I don’t even want to try to have somebody have me do things. And I just didn’t. I shied away from it. I was too scared. I mean, it’s a whole change in your life when something happens, and you don’t have the same mobility, and then you’re going to have somebody working with you. I don’t know. For me, it just scared me, so I held off, and I’ve continued to hold off for a year.”*

(003)

For those who did receive recommendations to increase PA, a notable discrepancy existed between healthcare providers’ suggestions and the participants’ comprehension of these recommendations and strategies for attaining PA goals. This disconnect led participants to question whether their needs could be adequately addressed:

*“...It’s obvious that there is a great need and not enough help.”*

(004)

Furthermore, participants expressed that available information concerning PA often lacked specificity tailored to the MS population. They also shared uncertainty regarding how to modify exercises and identify suitable activities. Half of the participants described negative experiences with PA shortly after the diagnosis, which contributed to heightened anxiety and fear. Lastly, participants struggled to determine the optimal level of PA and how to measure and track their progress—factors that significantly influenced their expectations and motivation for PA. Thus, PA participation appears to be difficult for people newly diagnosed with MS without an adequate education and understanding of their body’s changing limitations and needs, and common MS-related symptoms like Uthoff’s phenomenon, as one participant shared,

*“That would be great to hear that information like, Hey, look, you know, on occasion, if you get too hot. This is what’s gonna happen. Once you cool back down, it’s gonna disappear and it will. It’s not like it’s continued progression in your MS.”*

(009)

### 3.2. Theme 2: Reimagining Physical Activity Leads to Frustration

Negative perceptions associated with PA extended beyond the anticipated pain and fatigue typically linked to PA. Many participants reported feelings of shame and embarrassment related to their altered physical appearance and diminished capabilities. One participant expressed their frustration:

*“I’m kind of over it... I went from all to pretty much nothing, and I’ve been through several courses of physical therapy... but I get discouraged.”*

(004)

Participants frequently expressed a loss of competence, leading to frustration over their inability to maintain previous PA levels. As a key driver of motivation, this diminished sense of competence turned exercise into a reminder of their limitations rather than an opportunity for improvement. Moreover, participants frequently shared that their understanding of PA needed to evolve due to their newly diagnosed condition, as one participant noted the following:

*“I think I’ll have a very difficult time dealing with that mentally. So kind of like educating and looking forward versus being in the here and now of Oh, I wanna go play tennis because that’s my form of exercise. It’s just kind of like reevaluating your life, and where you’re at, and it may or may not be related to your age.”*

(010)

This shift in perception—grappling with the limitations of their diagnosis—led to considerable frustration and highlighted a threat to autonomy, with many individuals questioning the benefits of these new forms of PA.

*“Dr. __ recommended water aerobics... and again, I think I’m in the water, there’s less impact, but how is it helping? How is it benefiting me? I guess I will have to try it to see, but my attitude towards exercise now is negative, I guess because I don’t feel like I can do much because of the nerve pain and fatigue and stuff like that.”*

(003)

Overall, participants felt that both the physical and mental strain put on them by their new diagnosis led to frustration surrounding PA, leading to concern about the future.

*“Well, it could be your physical limitations. But also just your mental, because I’m concerned about myself moving forward. If it gets to the point where I can’t physically do what I’m doing now.”*

(010)

Highlighting a threat to relatedness, individuals often feared missing out on activities and worried about what the future might bring, as the prospect of physical decline threatened their sense of community.

### 3.3. Theme 3: Navigating PA Constraints in a Busy World

Individuals newly diagnosed with MS encountered numerous barriers to engaging in PA, often revolving around the challenge of finding time to commit. As a result, 50% (6/12) reported that these barriers currently outweighed their motivation to participate. However, 67% (8/12) of individuals reported maintaining a regular exercise routine, slightly higher than that observed in previous studies (41%; Table 1) [38]. Participants cited difficulties including physical activity into their busy schedules, which included work, childcare responsibilities, and household management:

*“[Regarding PA] I should, but I don’t. You know partly, maybe we’ll get into some of this, it’s time. I just don’t have time. Sometimes by the end of the day, you’re done with dinner and the dishwasher, and it’s like ’I’ve had it for the day’.”*

(001)

The lack of time, as expressed by participants, posed a significant challenge to autonomy, with external obligations taking precedence over PA and leaving them feeling a loss of control over their schedules. Many participants remained employed, and their work commitments often took precedence over engaging in physical activity, leaving them with limited energy reserves for exercise:

*“Time and fatigue level. Well, work is the priority. Things you got to do; you do. While this [PA] is in spare time, which there is never spare time.”*

(002)

This lack of choice contributes to a decline in motivation, stemming from the feeling of losing control over their actions. Although some participants managed to allocate time in the evenings for PA, they often encountered fatigue and pain after a demanding day, making them too exhausted to make effective use of this time:

*“...by that time of the day, which is really the time I have after work... from the impact of walking all day or just up and moving, by then it hurts so bad. It discourages me from doing anything, and then I just want to sit. Then I don’t want to be on it. Not to drive, not to walk on it. It’s just aggravating. I’m hurting. Then I do have the fatigue of course that I struggle with all day. So by then, when it’s time to exercise, it’s just not how it used to be. I don’t have the energy.”*

(003)

The perception of a lack of time during the day was frequently accompanied by the belief that increasing PA levels required specialized equipment or a gym membership, a sentiment shared by 50% of the participants. This, in turn, created a perceived barrier, as it introduced a monetary cost associated with PA and further exacerbated time constraints. This belief connects to both competence and autonomy. When individuals perceive that PA requires external resources like specialized equipment or a gym membership, it can create feelings of inadequacy regarding their ability to engage in PA without these resources.

### 3.4. Theme 4: Accountability Is Key to Maintaining PA in the Presence of Barriers

Participants shared successful personal strategies used currently or in the past but highlighted the challenges (Theme 3) to regular PA. Overall, participants expressed that support, whether from a partner or a group, was beneficial in increasing PA.

*“I would just think, having a partner to exercise with, to hold you accountable, hold each other accountable.”*

(012)

In this case, having an exercise partner fosters a sense of connection and accountability, promoting relatedness. While many people expressed time and accessibility as a barrier, the overall belief was that working in partnership with a physical therapist or medical professional toward PA goals was preferred.

*“[Regarding physical therapist or medical professional] So somebody who understands that (heterogeneity of MS) and knows the disease, but also gets to know the person and what their motivation is…somebody who is an actual professional who could sort of help guide in that way.”*

(006)

Professionals who understand the person’s needs and provide tailored guidance can boost confidence and perceived competence, making PA seem more achievable and less intimidating. The overall sentiment was that accountability, in whatever form, was essential to increasing PA. Specifically, individuals newly diagnosed with MS frequently mentioned the benefits of wearables, as they could increase accountability and gamify working out, allowing for tracking of PA and other health behaviors.

*“[Regarding wearables] It’s interesting. It is interesting to look and go, ’I didn’t do anything today.’ It goes off, and I’m like, ’oh I did my steps today.’ It’s a good feeling.”*

(002)

*“It’s really good for gamifying working out. It’s really good for also keeping track of steps it gives you an opportunity to make sure that you continue to drink water cause you can track water. So it’s really good at not only making a subjective, but also an objective, like view of your workout.”*

(009)

The use of wearables offers participants a sense of control over their PA—tracking their steps, monitoring hydration, and seeing real-time feedback about their exercise routines. This ability to track progress helps enhance their competence by providing measurable data that allow participants to assess their effectiveness in meeting their PA goals. Additionally, wearables contribute to autonomy by giving individuals more control over how they engage in PA. Participants can decide how to use the data to improve their routines, whether by setting goals, challenging themselves to increase steps, or adjusting their behaviors.

### 3.5. Logic Model for ENGAGE-MS

The findings of the study and the resulting themes highlight the existing gap in adherence to PA and its maintenance among individuals newly diagnosed with MS. Specifically, the study identifies themes that point to a lack of knowledge about PA and the challenges of adapting to a new diagnosis (Themes 1 and 2). Furthermore, participants in the study acknowledge the importance of strategies for managing time for exercise and maintaining accountability, as crucial factors in developing successful and sustained PA behaviors (Themes 3 and 4).

The identified themes informed the adaptation of a PA coaching intervention for individuals newly diagnosed with MS (ENGAGE-MS). The ENGAGE intervention was initially developed for individuals with Huntington’s disease [39], a complex neurodegenerative disease, and has been adapted for people newly diagnosed with Huntington’s and Parkinson’s [40]. ENGAGE is a PA coaching intervention delivered by physical or occupational therapists, with individualized coaching sessions grounded in self-determination theory. The ENGAGE program addresses barriers to exercise by targeting individuals early in the disease process to facilitate more physically active lifestyles, incorporating individualized goals, and promoting disease self-management. Specifically, the ENGAGE-MS coaches and the PA workbook provide suggestions and guidance for reimagining physical activity and identifying ways to tailor activities to an individual’s ability level. Compared to in-person delivery, remote interventions incorporating provider interaction can improve accessibility to self-management strategies by overcoming barriers such as cost, mobility restrictions, and limited service availability in rural and remote areas [40,41].

**Figure 1:**
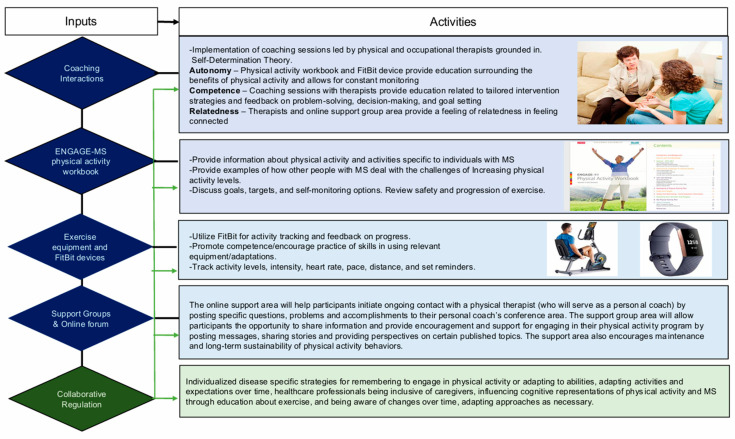
Logic model for ENGAGE-MS.

In Figure 1, we present a logic model for ENGAGE-MS. The key components of the ENGAGE-MS intervention include one-on-one coaching by a physical therapist, a disease-specific workbook developed based on the results of the focus groups, and the use of Fitbit (San Francisco, California) PA tracking devices for self-monitoring.

Based on the key themes identified, the ENGAGE workbook has been modified to include information regarding the pathology of MS and the impact that PA can have on the maintenance of function (Theme 1). To ensure inclusivity and generalizability, educational materials can be made available in both printed and online formats, with information being provided through videos and interaction with the coach (Theme 4). The workbook specifically discusses strategies to overcome the most common barriers identified from the focus groups (Theme 3).

Coaches will work with participants one on one to identify individual perceptions of exercise and overcome both barriers and misconceptions about PA (Themes 1, 2, and 3). As some participants in our study expressed negative feelings about PA, ENGAGE-MS will focus on activities that each individual likes to partake in, promoting autonomy and a range of options for staying active. The coach will work with each individual to identify tailored intervention strategies, which may include small bursts of PA, scheduled rest, and an emphasis on accountability.

## 4. Discussion

The significance of PA in individuals with MS is of paramount importance, as its implications have far-reaching effects, ultimately influencing overall quality of life [42]. However, recent evidence suggests that individuals with MS do not engage in a sufficient level of PA [6]. Furthermore, current research highlights the critical importance of increasing PA shortly after the diagnosis, as elevated levels of PA have the potential to slow or even halt the progression of the disease [43]. Additionally, a recent systematic review revealed that theory-based PA behavior change interventions have not adequately focused on individuals with MS within the first five years of the diagnosis [44]. These findings highlight the missed “window of opportunity” to enhance PA levels in individuals with MS, consequently increasing the likelihood of delaying clinical symptoms and postponing mobility decline [4,14]. Our findings address this knowledge gap by offering valuable insights into the barriers and facilitators of PA in individuals newly diagnosed with MS. They also identify potential targets to encourage increased PA and underscore the promise of PA coaching interventions like ENGAGE-MS.

Consistent with the previous qualitative literature concerning individuals with MS, our findings underscore the significance of education provided by healthcare providers and exercise professionals regarding PA levels and safety [19,20,45,46]. These results suggest that, in comparison to individuals who have been diagnosed with MS for a longer duration, those who are newly diagnosed with the condition encounter similar barriers related to their understanding of PA, its importance, and its overall implications for health-related outcomes. Overall, participants in our study reported a lack of knowledge regarding the importance of increasing PA, what activities were safe and beneficial for them, and what resources were available to support them. Furthermore, this knowledge and access gap appears to persist independently of disease severity, as individuals report these challenges whether they are newly diagnosed or have significant mobility impairments, as observed in the study by Van der Linden et al. [21]. The recurring theme of limited access to PA opportunities in qualitative research on PA barriers in individuals with MS highlights the urgent need for interventions aimed at bridging this gap [47].

Findings from our study underscore the significant role that pre-diagnosis perceptions of PA play in shaping subsequent PA behaviors. Notably, individuals’ perceptions of PA are heavily influenced by their pre-diagnosis abilities, resulting in frustration from changes to new circumstances, resulting in questions about the potential benefits of participating in PA. These findings contrast with previous studies in which preserving physical function was identified as a facilitator [2]. Additionally, differences emerged between the studies included in their review and the present study. Notably, newly diagnosed participants in our study did not mention environmental barriers, such as lack of accessibility. Instead, they emphasized a lack of education about PA for individuals with MS, as well as personal barriers like fatigue, influenced by societal and familial roles. This discrepancy is likely due to differences in the disease stage between the study populations. Our study included individuals in the early stages of MS, whereas the review did not specifically focus on newly diagnosed individuals. These findings highlight the unique barriers to physical activity faced by those recently diagnosed with MS and underscore the importance of education and adaptable PA modalities that align with personal lifestyles.

In contrast to the findings of Borkoles et al. [19], where participants indicated that they were no longer “bothered” by their limited abilities compared to before their diagnosis, participants in our study experienced significant frustration with their new situation, resulting in reduced PA. The disparity in findings may be attributed, in part, to the difference in time since the diagnosis between the Borkoles et al. study and ours. The participants in the prior study were, on average, 16.3 years post-diagnosis, whereas all participants in our study were on average within 3 years of the diagnosis. The reported decline in PA due to frustration observed in our study aligns with the previous literature identifying that individuals newly diagnosed with MS tend to employ fewer coping strategies crucial for adaptation [48]. Instead, these individuals are more likely to resort to avoidance strategies when confronted with a new and stressful situation [48]. Taken together, these findings underscore the importance of MS-specific PA programs, as increased social support and accountability play critical roles in enhancing self-efficacy [49]. Research has demonstrated that self-efficacy is crucial for sustaining or enhancing physical activity levels over time [50], and the ENGAGE program has been shown to significantly improve self-efficacy in participants with neurological disorders [39]. As seen in our study, participants identified that this support and accountability can take many forms, including a healthcare provider, a spouse, other individuals with MS, and wearables. As such, future interventions should seek to incorporate social support and accountability to boost self-efficacy for PA.

Participants in our study reported that difficulties in allocating time for PA resulted in reduced PA engagement, as societal roles took precedence. Specifically, individuals disclosed that employment presented challenges by both limiting available time and exacerbating pain and fatigue, thereby diminishing the likelihood of PA participation. These findings represent a novel contribution to the field, as prior qualitative research on barriers and facilitators to PA did not identify employment as a specific obstacle [2]. It is worth noting that these findings may pertain primarily to individuals recently diagnosed with MS, as previous research has established a connection between employment rates and disease duration [51]. The constraints of time and the burden of fatigue diminished the likelihood of individuals actively pursuing structured approaches to enhance their PA. Therefore, interventions that emphasize autonomy and flexible delivery methods could prove beneficial in raising PA levels among individuals newly diagnosed with MS.

This study and those previously conducted in individuals newly diagnosed with MS underscore the daily challenges associated with integrating PA into the lives of individuals recently diagnosed with MS. Our study and those of Huynh et al. emphasize the role of healthcare providers in providing support and education, thereby promoting self-efficacy [17,29]. Given the prominent role of time and employment as barriers for those with newly diagnosed MS, delivering this information remotely is likely to promote PA behaviors. The consistent findings across studies emphasize the crucial role of social support for individuals newly diagnosed with MS, highlighting the need for novel interventions that ensure that these individuals feel supported by both healthcare professionals and their communities. A novel aspect of our study is the use of wearables as a tool for tracking PA and promoting accountability, alongside the consistent identification, by participants, of fatigue from societal roles as a significant barrier to PA engagement. Collectively, the barriers to PA identified in this study underscore the importance of implementing a structured PA program, such as ENGAGE. The ENGAGE program is a well-established PA intervention, previously employed in other neurological conditions shortly after the diagnosis, with the primary objective of encouraging more active lifestyles [39,52]. Built on the principles of self-determination theory [39], ENGAGE integrates one-on-one coaching with a physical or occupational therapist, delivered through an online format, a workbook containing disease-specific information about the diagnosis and the importance of PA, and the utilization of Fitbit tracking to enhance accountability and provide additional insights into PA behavior [53]. Given that the ENGAGE intervention incorporates insights derived from participant perceptions of PA barriers, it offers a holistic approach to behavior change tailored specifically to the needs of individuals recently diagnosed with MS. Moreover, the adaptability of the ENGAGE intervention to an individual’s ability level and lived environment enables participants from diverse settings, including urban and rural areas, to benefit from it, as demonstrated in previous research [39]. As such, future research should explore the implementation of the ENGAGE intervention for individuals newly diagnosed with MS.

While our study boasts several strengths, it is necessary to acknowledge its limitations. First, the small sample size limits the generalizability of our findings, as the perceptions of our sample may not fully represent the broader population. Nevertheless, our study did achieve saturation, with common themes emerging after three focus groups that were confirmed with a fourth focus group. However, PA behavior is strongly influenced by various sociodemographic factors, including age, gender, socioeconomic status, and living environment. Therefore, the perspectives of those included in our focus group may not fully represent the broader MS population, as barriers to physical activity are both diverse and highly individual. Furthermore, participation in the study was voluntary, which may introduce biases related to barriers and facilitators. Additionally, the qualitative nature of our study does not allow for an in-depth understanding of the impact of both barriers and motivators on PA levels in this population.

## 5. Conclusions

The significance of PA for individuals recently diagnosed with MS is vital to long-term quality of life. Our study identifies both common barriers and facilitators shared by newly diagnosed individuals with MS and the broader population. Moreover, it reveals unique barriers faced by those recently diagnosed, notably employment. Healthcare providers should prioritize education and resource sharing on PA for individuals newly diagnosed with MS, with a particular focus on safe exercise modalities, strategies for managing symptoms like fatigue, and practical approaches to integrating physical activity into daily life. In light of our study’s results, the incorporation of interventions like ENGAGE-MS, which encompass education, accountability, and flexibility within the daily lives of newly diagnosed individuals with MS, may lead to increased PA levels. Our findings particularly highlight the significance of early education about PA and its short- and long-term benefits on quality of life, and our future studies will further explore efficacy of ENGAGE-MS in persons newly diagnosed with MS.

## Figures and Tables

**Table 1:** Participant Demographics.

Participant ID	Sex	Age (Years)	Race	Years Since Diagnosis	Years Since Symptoms	DMT (1 = Yes)	Regular Exercise (1 = Yes)	PDDS
001	M	56	C	4	5	1	1	3
002	F	56	C	3	3	1	0	0
003	F	49	C	1	1	0	0	1
004	F	65	C	1	1	1	1	4
005	F	63	AA	2	3	1	0	3
006	F	56	C	4	1	1	1	1
007	F	64	AA	5	11	1	0	4
008	M	57	C	2	5	1	1	4
009	M	35	M	4	11	1	1	0
010	F	40	C	4	4	1	1	0
011	F	38	C	4	20	1	1	2
012	F	53	AA	3	3	1	1	1
Mean (±SD)	3 M 9 F	52.67 ± 10.18	67% C 25% AA 9% M	3.08 ± 1.31	5.67 ± 5.66	11/12 91.67%	8/12 66.67%	1.92 ± 1.62

Male (M); Female (F); African American (AA); Caucasian (C); Multiracial (M); Disease-Modifying Therapy (DMT); Patient-Determined Disease Steps (PDDSs).

## Data Availability

Codes supporting the conclusions of this article will be made available by the authors upon reasonable request.

## Appendix A. Focus Group Questions

(1) What do you think about when you hear the terms “Exercise for MS”? If needed, an additional prompt was provided: Do you think it is something that could be helpful for management of MS?
(2) What would you have liked to know about exercise or physical activity when newly diagnosed or early in the course of MS?
(3) Compared to other things in your life, where does exercise fit in?
(4) Even if it wasn’t easy to do, what would help you to adapt to a new routine focusing on exercise?
(5) One way to monitor improvements with activity levels or exercise is with wearable devices, such as Fit-Bits. What do you think about this?
(6) For this question, individuals were shown a mock-up of an exercise workbook that was initially developed for persons with Parkinson’s disease and asked to give their feedback with the following question: We are developing a program specifically for persons with MS who are newly diagnosed with a goal of increasing physical activity. In this program, individuals would work with a coach and utilize this booklet. What do you think of this workbook?
